# Postpartum Depression in High-Risk Pregnancies: Findings from a Telepsychiatry-Based Longitudinal Follow-Up

**DOI:** 10.3390/jcm15145405

**Published:** 2026-07-10

**Authors:** Fatma Seher Kocaayan, Beyza Türk, Elif Tatlıdil, Aslıhan Polat

**Affiliations:** 1 Department of Psychiatry, Kocaeli University Research and Application Hospital, 41001 Kocaeli, Türkiye; seherkocaayan@gmail.com (F.S.K.); beyzaturkk@gmail.com (B.T.); 2 Department of Psychiatry, Sakarya Training and Research Hospital, 54100 Adapazari, Türkiye

**Keywords:** postpartum depression, high-risk pregnancy, telepsychiatry, social support, fetal anomaly, longitudinal study

## Abstract

**Highlights:**

**What are the main findings?**

- PPD was identified in 32.7% of this selected high-risk pregnancy cohort during the first two postpartum months.
- Lower marital satisfaction, lower perceived social support, lower spouse education, and fetal anomaly were associated with PPD in the exploratory multivariable model.

**What are the implications of the main findings?**

- The identification of additional new-onset PPD cases at the second-month assessment supports the importance of repeated postpartum mental health assessment rather than relying on a single early screening.
- Telepsychiatry-based follow-up appears to be a feasible approach for postpartum mental health assessment and may support the early identification of PPD in women with high-risk pregnancies.

**Abstract:**

**Background/Objectives:** Postpartum depression (PPD) is a significant public health issue, particularly in high-risk pregnancies where medical complexities increase maternal vulnerability. This study aimed to determine the prevalence of PPD in a high-risk parturient population and to explore factors associated with PPD using a telepsychiatry-based longitudinal follow-up model. **Methods:** This longitudinal pilot study followed 58 women who met high-risk pregnancy criteria at a university hospital in Turkey. Initial assessments were conducted face-to-face within 72 h post-delivery, followed by telepsychiatric evaluations at the first and second months postpartum. PPD was diagnosed via structured clinical interviews based on DSM-5 criteria, supplemented by the Edinburgh Postnatal Depression Scale (EPDS) and the Multidimensional Scale of Perceived Social Support (MSPSS). Logistic regression was used to explore factors associated with PPD. **Results:** The cumulative prevalence of PPD during the first two postpartum months was 32.7%: 13 participants (22.4%) were diagnosed at month 1, and 6 additional new-onset cases (10.3%) were identified at month 2. In the exploratory multivariable logistic regression model, low marital satisfaction (OR = 26.13, *p* = 0.005), lower postpartum MSPSS total scores (OR = 0.90, *p* < 0.001), the presence of a fetal anomaly (OR = 15.10, *p* = 0.043), and a lower spousal education level (OR = 17.19, *p* = 0.014) were associated with PPD. However, these estimates should be interpreted cautiously because of the limited number of PPD events and wide confidence intervals. Notably, comorbid medical conditions and stressful life events during pregnancy did not remain significant in the final model. **Conclusions:** The observed PPD prevalence in this selected high-risk cohort was higher than previously reported general-population estimates. Psychosocial factors, particularly marital satisfaction and perceived social support, showed strong associations with PPD in this pilot cohort. A fetal anomaly may also represent an important clinical risk marker, but this finding requires confirmation in larger samples. A telepsychiatry-based follow-up model appears feasible for postpartum mental health assessment in this vulnerable group and may help support the early identification of PPD.

## 1. Introduction

Postpartum depression (PPD) is a common psychiatric disorder that negatively affects maternal well-being and early childhood development. With a reported prevalence of approximately 24% in Turkey, it represents a significant public health concern [1], and a significant public health concern [2].

Having a medical complication during pregnancy and childbirth is a key risk factor for PPD. Women with pregnancies complicated by fetal anomalies, maternal illness, or advanced maternal age constitute a clinically vulnerable group requiring close monitoring [3,4]. Although there is a higher incidence of PPD among women with high-risk pregnancies, the mental health screening and longitudinal follow-up model in a tertiary care setting is underdeveloped [5].

If not treated, PPD may lead to major consequences like maternal dysfunction, developmental delays in children, and increased suicide risk [6,7,8]. The condition’s clinical importance is often overshadowed by its stigma; many women do not mention their condition to professionals for fear of a negative diagnosis relating to motherhood [9,10]. Obstacles such as logistical hurdles and heavy clinical workloads in tertiary centers further impair timely access to psychiatric assessments.

Telepsychiatry has emerged as a promising alternative to overcome these hurdles by enabling remote access to care [11]. Nonetheless, there is a lack of research regarding telepsychiatry for systematic PPD screening of high-risk obstetric cases. The objective of this research is to assess the prevalence of PPD among high-risk parturients attending a university hospital as well as to examine the relationship between depressive symptoms, social support, and obstetric parameters with a view to exploring factors associated with postpartum depression.

## 2. Methods

### 2.1. Study Design and Sample

This pilot study was conducted within the framework of the Consultation–Liaison Psychiatry (CLP) telepsychiatry postpartum follow-up program carried out at a tertiary university hospital. This program aims to clinically evaluate all women who give birth during the postpartum period, provide psychoeducation, and follow up with the cases that receive a diagnosis. In this study, it aimed to reach all women who gave birth at the university hospital between October 2025 and December 2025. During the study period, 152 of the 156 women who gave birth agreed to participate in the study, and sociodemographic data were collected from these participants during the postpartum period.

**Figure 1:**
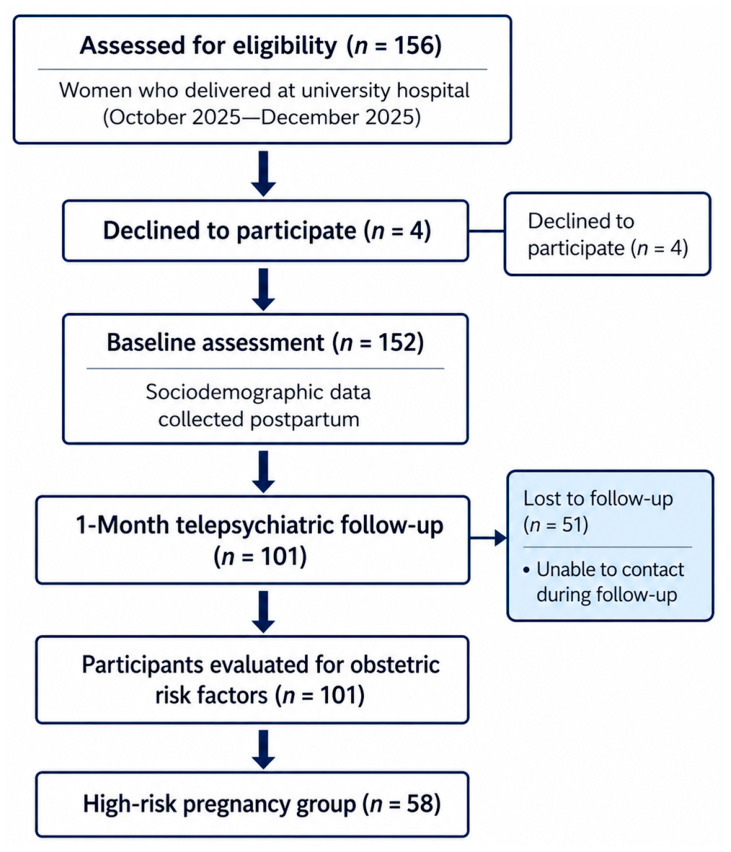
Flow diagram of participant recruitment, telepsychiatric follow-up and final analysis.

A telepsychiatry follow-up interview was conducted with 101 participants in the first postpartum month, while 51 participants could not be contacted. In order to determine the main analysis group of the study, the 101 participants who completed the first postpartum month interview were evaluated in terms of obstetric risk factors. Among these women, 58 who had at least two of the following risk parameters—presence of a health problem during pregnancy, maternal comorbidity, a fetal anomaly, advanced maternal age (≥35), obesity (BMI ≥ 30), or a history of preterm birth—were classified as having “high-risk pregnancy.” Since the literature shows that an increasing number of risk factors is associated with a higher risk of PPD, a threshold of “at least two risk factors” was used in this study to increase specificity [12]. The study was conducted on a selected sample focusing on the high-risk pregnancy group, and the final analyses were performed with participants who met the predefined criteria for high-risk pregnancy (Figure 1).

This definition was used as an operational study definition rather than as a universally established international classification system. The threshold of at least two risk factors was selected to identify a subgroup with a higher cumulative obstetric risk burden and to avoid classifying women with only a single, relatively common risk factor as high-risk. All risk factors were treated as binary variables and were not weighted according to clinical severity, because the sample size of this pilot study did not allow the construction or validation of a weighted obstetric risk score.

High-risk pregnancy is a clinical classification that refers to pregnancies with an increased likelihood of complications for the mother and/or the newborn. Although there is no complete consensus in the literature regarding its definition, factors such as advanced maternal age, maternal comorbidities, obesity, fetal anomalies, and a history of preterm birth are commonly used determinants of a high-risk pregnancy. In addition, the presence of multiple obstetric risk factors together has been shown to be more strongly associated with adverse pregnancy outcomes [13,14]. For this reason, the definition of high-risk pregnancy in this study was based on commonly used obstetric risk determinants, while acknowledging that the ≥2 risk-factor threshold represents a pragmatic operational criterion for this pilot study rather than a validated risk classification.

Since PPD has been reported to occur most frequently during the first weeks after childbirth and especially within the first three months, two separate assessments were conducted within the first two months postpartum in this study [15,16]. Participants who received a DSM-5-based clinical diagnosis of PPD at either the first-month or second-month assessment were included in the cumulative PPD group. Cases detected at the second-month assessment refer to additional new-onset PPD cases among participants who had not received a PPD diagnosis at the first-month assessment.

The data collection was a multi-stage verification process. Before the tele-interview, the EPDS and MSPSS scales were sent to the participants through digital means. Subsequently, a structured clinical interview was conducted by a psychiatrist via telepsychiatry for all participants. A diagnosis of PPD was not determined solely on the basis of self-report scale scores; rather, all diagnoses were clinically confirmed by a psychiatrist through a structured interview based on DSM-5 criteria for a major depressive episode. According to the principle of continuity of treatment, participants with diagnosis were admitted to regular treatment and control program via the same system.

To assess potential attrition bias, baseline sociodemographic, psychiatric, and obstetric characteristics were compared between participants who completed the first-month telepsychiatric follow-up and those who were lost to follow-up.

### 2.2. Inclusion and Exclusion Criteria

The study’s inclusion criteria were set as being 18 years or older, hospitalization in the first 72 h after delivery, the ability to read and understand Turkish, and possessing the necessary digital communication tools for telepsychiatric follow-up. For the longitudinal follow-up phase of the study, the selection of study participants was limited to women who completed the first postpartum month telepsychiatry interview, had at least two obstetric risk factors, and completed all assessments. The final analysis population comprised 58 participants who met these criteria.

The exclusion criteria were defined as the presence of active psychotic symptoms, severe cognitive impairment, incomplete or incorrectly completed scales, and the presence of a clinically unstable medical condition.

### 2.3. Data Collection Process and Telepsychiatry Application

The data collection process was conducted using a three-stage follow-up model. In the first stage, all women who gave birth were evaluated face-to-face by the CLP team during hospitalization within the first 72 h postpartum. After obtaining informed consent, participants completed the “Sociodemographic and Clinical Information Form,” which included sociodemographic and clinical characteristics, and their contact information was recorded for telepsychiatric follow-up. Obstetric and medical data such as a fetal anomaly, physical illness during pregnancy, comorbid medical conditions, a history of preterm birth, and obesity were recorded based on medical records obtained from the hospital information system rather than participant self-report in order to increase data reliability.

In the second stage, participants were evaluated in the first postpartum month (±1 week) using a telepsychiatry method. All telepsychiatry interviews were conducted by the same psychiatrist to ensure intra-rater consistency. Before the interviews, participants were sent the Edinburgh Postnatal Depression Scale (EPDS) and the Multidimensional Scale of Perceived Social Support (MSPSS) through digital platforms and were asked to complete the scales online.

In the third stage, participants were invited to a telepsychiatric follow-up interview in the second postpartum month (±1 week). At this stage, after administering the EPDS, a structured clinical interview was conducted via telepsychiatry, and a diagnosis of PPD was evaluated based on the DSM-5 diagnostic criteria for a major depressive episode.

### 2.4. Data Collection Instruments

Three different data collection instruments were used in the study to evaluate the sociodemographic, clinical, and psychosocial characteristics of the participants.

#### 2.4.1. Sociodemographic and Clinical Information Form

This form was prepared by the researchers and was used to assess the participants’ sociodemographic and clinical characteristics, including age, education level, economic status, employment status, history of psychiatric illness, and partner relationship.

#### 2.4.2. Edinburgh Postnatal Depression Scale (EPDS)

This is a self-report scale consisting of 10 items used to screen for symptoms of PPD. Each item is scored between 0 and 3, and the total score ranges from 0 to 30 [17]. In the Turkish validity and reliability study of the scale, the cutoff score was determined as 13, and the same cutoff value was used in this study [18].

#### 2.4.3. Multidimensional Scale of Perceived Social Support (MSPSS)

This is a 12-item self-report scale used to assess the level of perceived social support. The scale consists of three subdimensions: family, friends, and a significant other, and each item is rated on a 7-point Likert-type scale. Higher scores obtained from the scale indicate a higher level of perceived social support [19]. The Turkish version, validated by Eker and Arkar (2001), confirmed the three-factor structure with a Cronbach’s alpha of 0.85 [20].

### 2.5. Statistical Analysis

Data analysis was performed using IBM SPSS Statistics v27.0 software (IBM Corp., Armonk, NY, USA). The distribution of continuous variables was evaluated using the Shapiro–Wilk test. Student’s *t*-test was used for variables that showed a normal distribution, while the Mann–Whitney U test was used for variables that did not show a normal distribution. Categorical variables were compared using the chi-square test or Fisher’s exact test.

Multivariable logistic regression was used to explore factors associated with the presence of PPD. Each variable that was statistically significant in the univariate analyses and/or deemed clinically relevant was included in the regression model. The VIF and standard error values were checked to assess multicollinearity. The fitness of the model was assessed by Hosmer–Lemeshow. The analysis excluded observations with insufficient data, and a listwise deletion approach was used in the analyses. A re-analysis was done on a total of 58 participants who fulfilled the standards for a high-risk pregnancy and completed all evaluations. A *p*-value less than 0.05 was considered significant. For scale comparisons across the three PPD groups (PPD−, Month-1 PPD+, and Month-2 PPD+), Kruskal–Wallis tests were conducted, followed by pairwise Mann–Whitney U post hoc comparisons with Bonferroni correction (adjusted significance threshold: *p* < 0.017).

### 2.6. Power Analysis

The study sample size was established through an a priori power analysis using G*Power software version 3.1.9.7 (Heinrich Heine University Düsseldorf, Düsseldorf, Germany). The calculation of the minimum sample size based on medium effect size (f^2^ = 0.15), α = 0.05, and 80% statistical power (1 − β = 0.80) indicated a minimum sample size of 55 to conduct a logistic regression model that included seven independent variables. Taking data loss into account, a sample size of 61 was applied. Final analyses were conducted with 58 participants who met the criteria for a high-risk pregnancy and fully completed all assessments while preserving the study’s planned statistical power level.

## 3. Results

As a preliminary step, baseline characteristics were compared between participants who completed the first-month follow-up (*n* = 101) and those lost to follow-up (*n* = 51). The two groups were broadly comparable with respect to age, education level, employment status, socioeconomic status, marital satisfaction, psychiatric history, and obstetric variables, including obesity, a preterm birth history, and advanced maternal age. The only statistically significant difference was a lower prevalence of comorbid medical conditions among participants lost to follow-up (*p* = 0.045), suggesting that the analyzed cohort may carry a slightly higher medical burden than the original sample.

Fifty-eight married, high-risk parturients were enrolled (mean age: 30.12 ± 5.84; mean gravidity: 2.36 ± 1.48; mean parity: 1.91 ± 0.94). Education levels ≤ 12 years predominated among the participants (63.8%) and their spouses (60.3%). Most participants were housewives (69%) with a low economic status (69%), whereas 94.8% of the spouses were employed. High marital satisfaction was reported by 65.5%. The mean one-month MSPSS was 66.90 ± 20.24 (Table 1).

Clinical and obstetric variables included medical comorbidities (25.9%), obesity (37.9%), psychiatric history (32.8%), smoking (19%), IVF (8.6%), and preterm birth (8.6%). Advanced maternal age (19%), fetal anomalies (19%), and delivery complications (17.2%) were observed. Gestational health issues (72.4%) and stressful life events (39.7%) were frequent. (Table 2).

At the first-month assessment, 13 participants (22.4%) received a DSM-5-based diagnosis of PPD. At the second-month assessment, six additional new-onset cases (10.3%) were identified among participants who had not received a PPD diagnosis at the first-month assessment. Thus, the cumulative prevalence of PPD during the first two postpartum months was 32.7% (19/58). This cumulative PPD variable was used for subsequent group comparisons and regression analyses. (Table 3).

Scale scores differed significantly across the three groups at both assessment points (Table 4). At the first-month assessment, EPDS total scores were markedly elevated in the Month-1 PPD+ group (median: 17) relative to both the PPD− group (median: 0; Z = −5.608, *p* < 0.001) and the Month-2 new-onset group (median: 3; Z = −3.437, *p* < 0.001), whereas the PPD− and Month-2 PPD+ groups did not differ significantly (Z = −1.138, *p* = 0.255). This pattern indicates that participants subsequently diagnosed at month 2 did not yet meet symptomatic thresholds at month 1, supporting the validity of the longitudinal screening design. For MSPSS total scores, significant differences were observed between the PPD− and Month-1 PPD+ groups (Z = −4.685, *p* < 0.001) and between the PPD− and Month-2 PPD+ groups (Z = −2.476, *p* = 0.013), both surviving the Bonferroni correction. The difference between the Month-1 PPD+ and Month-2 PPD+ groups did not survive the correction (Z = −2.326, *p* = 0.020), suggesting broadly comparable social support deficits across both PPD subgroups relative to the non-PPD group.

At the second-month assessment, all three pairwise comparisons for EPDS total scores were statistically significant after the Bonferroni correction (PPD− vs. Month-1 PPD+: Z = −4.732, *p* < 0.001; PPD− vs. Month-2 PPD+: Z = −4.485, *p* < 0.001; Month-1 PPD+ vs. Month-2 PPD+: Z = −2.907, *p* = 0.004). The Month-1 PPD+ group showed a decline in the median EPDS score from 17 at month 1 to 9 at month 2, consistent with partial symptomatic improvement following treatment initiation within the telepsychiatry follow-up program. Formal remission criteria were not applied; therefore, complete symptomatic recovery cannot be confirmed from scale scores alone. Formal diagnostic accuracy analyses, including sensitivity, specificity, and the area under the receiver operating characteristic curve, were not performed.

The PPD (*n* = 19) and non-PPD (*n* = 39) cohorts differed significantly regarding spousal education (*p* = 0.043) and marital satisfaction *p* = 0.009). No significant differences (*p* > 0.05) existed for age, gravidity, parity, maternal education, employment, economic status, or infant sex. Conversely, the PPD cases had significantly lower first-month MSPSS total and subdimension scores (*p* < 0.001). Median total scores were 51 (12–78) vs. 84 (17–84) (*p* < 0.001); the significant other (*p* < 0.001), family (*p* < 0.001), and friends (*p* = 0.001) subdimensions were also significantly reduced (Table 5).

Clinical and obstetric variables—including comorbidities, psychiatric history, and delivery complications—showed no significant differences (*p* > 0.05), despite numerically higher rates of advanced age and fetal anomalies in the PPD group (Table 6).

A logistic regression analysis was conducted to evaluate factors associated with PPD. The MSPSS total score, spousal education level, and marital satisfaction, which were found to be significant in the bivariate analyses, were included in the model. In addition, the variables stressful life event during pregnancy, comorbid medical condition, and fetal anomaly were added to the model because they were considered clinically relevant.

According to the Omnibus Test of Model Coefficients, the overall model was statistically significant (χ^2^(6) = 37.829; *p* < 0.001). The explanatory power of the model was calculated as Cox and Snell R^2^ = 0.479 and Nagelkerke R^2^ = 0.668. The Hosmer–Lemeshow goodness-of-fit test indicated that the model showed a good fit to the data (χ^2^(8) = 4.972; *p* = 0.761). The overall classification accuracy of the model was 84.5%, correctly classifying 87.2% of non-PPD cases and 78.9% of PPD cases.

According to the logistic regression analysis, marital satisfaction (B = 3.263; *p* = 0.005; OR = 26.129; 95% CI: 2.623–260.232), the MSPSS total score (B = −0.106; *p* < 0.001; OR = 0.900; 95% CI: 0.846–0.957), the presence of a fetal anomaly (B = 2.714; *p* = 0.043; OR = 15.095; 95% CI: 1.092–208.666), and the spousal education level (B = 2.844; *p* = 0.014; OR = 17.185; 95% CI: 1.760–167.755) were associated with PPD. In contrast, the presence of a comorbid medical condition (*p* = 0.399) and a stressful life event during pregnancy (*p* = 0.334) were not found to be significant (Table 7).

## 4. Discussion

This study evaluated PPD prevalence and its clinical correlates among 58 high-risk parturients in a tertiary hospital. Our cohort represented a medically complex group, characterized by notable rates of comorbidities (25.9%), fetal anomalies (19%), and a prior psychiatric history (32.8%). Most participants were around 30 years old, with over 60% having an education level of 12 years or less, further underscoring the population’s vulnerability.

The cumulative prevalence of PPD during the first two postpartum months reached 32.7%, exceeding the 17.22% global mean [2] and the 24% Turkish average [1]. This elevation stems from the high-risk cohort. Perinatal depression reaches 39% in hospitalized high-risk patients, as antenatal distress persists postpartum [21,22]. While restricting generalizability, these data isolate risks within a clinically vulnerable population.

Screening scales yield more false positives than clinical interviews [23,24]. Postpartum stigma drives underreporting [25,26]. Structured clinical confirmation likely explains why our prevalence exceeded screening data. PPD onset concentrates within the first three postpartum months [15,16]. Two assessments during the first two months identified early cases. Telepsychiatry improves early detection by expanding screening reach [27].

In addition to the study population consisting of high-risk pregnancies, the high proportion of certain sociodemographic characteristics in the sample is also notable, including low education level (63.8%), being a housewife (69%), and a poor economic status (69%). These characteristics largely overlap with the sociodemographic risk factors identified in the literature for PPD [28,29].

Fetal anomalies, delivery complications, gestational stress, advanced maternal age, and comorbidities were more prevalent in the PPD group without reaching statistical significance. The universal high-risk status likely obscured intergroup differences by elevating baseline obstetric risk across the cohort. The limited sample size also constrained statistical power.

According to the results of our study, the spousal education level, marital satisfaction and perceived social support—which were variables that were significant in the bivariate analyses—remained significantly associated with PPD in the regression model. In addition, a fetal anomaly was associated with PPD in the exploratory model, although this finding should be interpreted cautiously because of the wide confidence interval and the limited number of PPD events. These findings are consistent with the literature indicating that partner relationship quality and social support are among the key determinants of PPD [30,31,32,33]. Although studies have not consistently demonstrated a direct association between spousal education level and PPD, some findings suggest that higher paternal education is associated with greater perceived social support for the mother [34]. This may indicate that a lower spousal education level could increase the risk of PPD by limiting communication and emotional support capacity.

The clearer association observed in the Turkish context may also be related to gender roles and the family structure. Studies on time use and the division of household labor in Turkey show that childcare and household responsibilities are largely undertaken by women, and caregiving is still predominantly perceived as “a woman’s responsibility” [35,36]. In this context, the spousal education level and the quality of the partner relationship become important determinants directly influencing the mother’s daily experience of support. It has been reported that partners with higher education levels may have greater health literacy, stronger communication skills, and a higher level of participation in the caregiving process, whereas more traditional role patterns and limited supportive behaviors may be more common at lower education levels [33]. Therefore, the significance of spousal education in this study suggests that postpartum mental health is shaped not only by individual factors but also by sociocultural dynamics related to the distribution of roles within the family and the structure of social support [37,38].

The link between partner relationship quality and depression is not limited to postpartum depression only. Dissatisfaction in marital or partner relationships has also been shown to increase the likelihood of experiencing major depressive episodes in the general population [39]. The study’s finding that relationship quality was lower in the PPD group, and that the partner relationship showed an association with PPD in the logistic regression model, points to the bidirectional nature of the interaction between depression and interpersonal relationships.

These findings may also be interpreted within the broader framework of postpartum psychosocial well-being. Postpartum mental health is not limited to depressive symptoms alone; it is closely intertwined with relationship quality, perceived partner support, family functioning, sexual well-being, and the mother’s overall adaptation to new parental roles. Brezeanu et al. recently emphasized that postpartum sexual health is an important component of the overall quality of life and relationship stability, and the postpartum sexual quality of life may be influenced by sociodemographic and obstetric factors, including the educational level, maternal age, parity, and the mode of delivery [40]. In this context, our findings regarding marital satisfaction, the spousal education level, and perceived social support suggest that partner-related and social support variables may reflect broader relational determinants of postpartum adjustment rather than factors related only to depressive symptoms.

The longitudinal scale comparisons further support these findings. At the first-month assessment, the MSPSS total and subdimension scores were significantly lower in the Month-1 PPD+ group relative to the PPD− group across all pairwise comparisons surviving the Bonferroni correction. The month-2 new-onset group showed intermediate MSPSS scores, an exploratory pattern suggesting that lower perceived social support may be detectable before a clinical PPD diagnosis in some women. Given the small subgroup size and the observational design, this finding should be interpreted cautiously.

In our study, lower perceived social support was significantly associated with PPD. The PPD group had lower MSPSS total and subdimension scores, and the first-month postpartum MSPSS total score remained associated with PPD in the exploratory regression model. However, the temporal relationship between perceived social support and PPD should be interpreted cautiously. In this study, the MSPSS scores were assessed during the postpartum follow-up rather than during pregnancy. Therefore, lower perceived social support may represent a pre-existing vulnerability factor for PPD, but it may also reflect the influence of depressive symptoms on the mother’s perception of available support. The association between social support and PPD is likely bidirectional: insufficient support may increase emotional vulnerability, while depressive symptoms may lead mothers to perceive existing support as less adequate. For this reason, our findings should be interpreted as an association rather than as evidence of a causal relationship. Future studies should assess social support longitudinally from pregnancy through the postpartum period to clarify temporal directionality.

In Turkey, postpartum cultural practices such as the “forty days” custom and ensuring that the mother is not alone may help protect the mother and baby by increasing social support. On the other hand, the weakening of support networks may increase psychological vulnerability. In addition, reduced social support due to migration and nuclear family structures has also been associated with postpartum depressive symptoms [41,42].

The birth of an infant with a fetal anomaly can represent a significant source of stress during the postpartum period due to complex medical needs, uncertainty regarding the prognosis, and demanding parental responsibilities. Previous studies have reported that PPD is more frequently observed among mothers of infants with congenital anomalies and that the severity of maternal depressive symptoms may be associated with the severity of the anomaly [43,44,45,46]. In our study, the finding that fetal anomaly showed an association with PPD suggests that this condition should be considered not only as an obstetric or neonatal problem but also as a major psychosocial stressor.

The psychological impact of a fetal anomaly may vary according to the timing of diagnosis and the subsequent neonatal course. When a fetal anomaly is diagnosed during pregnancy, the mother may experience uncertainty, guilt, anticipatory grief, anxiety, and traumatic stress beginning in the antenatal period, which may persist into the postpartum period and contribute to depressive symptoms [47,48,49]. In contrast, anomalies that are detected after delivery or become clinically evident during the neonatal period may impose a different psychological burden, related to unexpected medical uncertainty, neonatal hospitalization, invasive interventions, possible surgical procedures, long-term caregiving demands, and concerns about the developmental prognosis. Therefore, the association between a fetal anomaly and PPD may reflect both an acute traumatic response to the diagnosis itself and the ongoing emotional strain associated with postnatal care needs. These clinically distinct pathways highlight the importance of early psychosocial assessment and consultation–liaison psychiatric support for mothers facing fetal or neonatal anomalies.

In contrast, stressful life events during pregnancy and comorbid medical conditions were not found to be statistically significant in the regression model. However, the literature frequently reports these variables as important risk factors for PPD [37,50,51,52,53,54,55,56]. The lack of statistical significance for these variables in our study may be related to the limited sample size and the generally high prevalence of obstetric risk factors within the sample. In addition, the dominant role of strong psychosocial variables such as social support and partner relationship in the model may also have influenced this result.

PPD has clinical importance not only because of its frequent occurrence but also due to the economic cost and mortality risk of the condition. Research by Luca et al. (2020) [57] indicates that if perinatal psychiatric disorders go untreated, they create major costs for society from pregnancy through early childhood. For this reason, screening for depression during the postpartum period is suggested. Screening scales are not diagnostic tools, and the results need to be verified by clinical interviews [24]. However, research indicates that many of the women identified as at risk through screening experience difficulty obtaining a psychiatric evaluation and treatment and are disrupted in the referral process [58]. Telepsychiatric models have been reported to improve service utilization through greater continuity of care and reduction in costly visits to healthcare facilities. Such approaches may have not only clinical benefits but also an economic benefit in perinatal mental health services by lowering barriers to access [59].

Telepsychiatry has emerged as an important tool in the field of PPD for overcoming structural barriers that limit new mothers’ access to mental health services. Factors such as concerns about stigma, time constraints, childcare responsibilities, and transportation difficulties are known to make access to face-to-face healthcare services more challenging. Telepsychiatry reduces these barriers by allowing care to be received from the home environment and thereby increases accessibility to services [11,60]. Indeed, the effectiveness of telepsychiatric interventions in the treatment of PPD has been supported by several systematic reviews and meta-analyses [61,62,63]. In high-risk pregnancy populations, such as those in our study, it is even more clinically important to enable flexible and sustainable access to mental health support. In the present study, however, there was no comparison group receiving conventional face-to-face follow-up. Therefore, our findings should not be interpreted as evidence that telepsychiatry is superior to or more effective than standard care. Rather, this study supports the feasibility of using a telepsychiatry-based follow-up model to conduct structured postpartum mental health assessments in a high-risk pregnancy population within a tertiary care setting.

From an implementation perspective, the existing telepsychiatry infrastructure in our center, which has been in use since the COVID-19 pandemic, facilitated the delivery of remote psychiatric assessments in this study. However, the postpartum follow-up model was specifically structured within the scope of the present study rather than being a pre-existing postpartum program. Therefore, the primary implementation challenge was not the conduct of telepsychiatric interviews per se, but ensuring consistent post-discharge contact and maintaining appointment continuity. Participants were informed before discharge about the follow-up process and the scheduled interviews. Video interviews were preferred whenever possible; however, participants who could not be reached by video call, experienced technical difficulties, or were unavailable at the initial contact attempt were contacted again by telephone, and new appointments were arranged according to their availability. Interviews that could not be completed because of technical problems were rescheduled. This flexible scheduling approach suggests that telepsychiatry-based postpartum mental health assessment is feasible in tertiary centers with an established telepsychiatry infrastructure, provided that systematic appointment coordination, repeated contact attempts, and alternative communication methods are incorporated into the follow-up model.

Postpartum clinical sensitivity and mortality risks necessitate early therapeutic engagement. Telepsychiatric interviews provide diagnostic capacity, psychoeducation, and prompt interventions. Continuity-based care models in high-risk populations optimize detection and management within postpartum mental health services.

### 4.1. Strengths

One of the main contributions of this study to the literature is its focus on a high-risk pregnancy population followed in a tertiary care center rather than the general obstetric population. The longitudinal design made it possible to identify cases of PPD that emerged within the first two months postpartum.

From a methodological perspective, one of the strongest aspects of the study is that the diagnosis of PPD was not based solely on self-report scales; instead, all assessments were confirmed through structured clinical interviews conducted by the same psychiatrist. This approach increased diagnostic accuracy while maintaining intra-rater consistency. In addition, data related to fetal anomalies and obstetric complications were obtained from hospital records rather than participant self-report, which reduced the risk of recall bias.

The use of an existing telepsychiatry infrastructure facilitated the implementation of remote follow-up assessments and supports the practical applicability of this model in tertiary care settings. Although the sample size was limited, the relatively homogeneous composition of the sample consisting of high-risk pregnancies, and the use of multidimensional data obtained from objective sources support the internal validity of the findings.

### 4.2. Limitations

Despite the clinical significance of our findings regarding PPD monitoring in high-risk pregnancies, several limitations must be acknowledged. First, the modest sample size (*n* = 58) constrained the statistical power of the study, resulting in wide confidence intervals for certain regression estimates. Specifically, the odds ratios for fetal anomaly (OR = 15.10; 95% CI: 1.09–208.67) and the spousal education level (OR = 17.19; 95% CI: 1.76–167.76) demonstrated considerable instability, necessitating caution in their interpretation. Consequently, these particular estimates should be viewed as preliminary and hypothesis-generating rather than definitive effect sizes for the broader high-risk population.

Second, the single-center design and the inclusion of a specific cohort within a tertiary care setting may limit the generalizability of our findings to low-risk or general obstetric populations. Future multicenter studies with larger, more diverse cohorts are warranted to validate these associations and refine our understanding of the factors contributing to PPD in high-risk pregnancies.

Attrition during follow-up represents another limitation. Although participants who completed the first-month follow-up and those lost to follow-up were broadly comparable across baseline sociodemographic, psychiatric, and obstetric characteristics, comorbid medical conditions were less frequent among those lost to follow-up. This difference suggests that selective attrition cannot be completely excluded and may have resulted in a follow-up cohort with a somewhat greater medical burden. Nevertheless, because no significant differences were observed across the other measured baseline characteristics, the extent of any observable selection bias appears limited. Unmeasured differences between responders and non-responders, however, cannot be ruled out.

Another limitation concerns the operational definition of a high-risk pregnancy used in this study. Although the selected risk factors were based on commonly recognized obstetric and maternal risk determinants, the threshold of at least two risk factors was not derived from a validated international classification system. Moreover, all risk factors were treated equally, despite potentially different clinical implications. For example, a fetal anomaly, maternal comorbidity, obesity, advanced maternal age, and a previous preterm birth may not carry the same level of obstetric or psychological risk. Therefore, the findings should be interpreted within the context of this pragmatic pilot-study definition. Future studies should consider using validated or weighted obstetric risk classifications in larger samples.

In addition, the multivariable logistic regression model may have been affected by overfitting because six predictors were included despite there being 19 PPD events. The low events-per-variable ratio and the wide confidence intervals for several odds ratio estimates indicate limited model stability and precision. Therefore, the regression findings should be interpreted as exploratory and hypothesis-generating rather than as a definitive predictive model. Larger studies are needed to validate these associations using more parsimonious models or penalized regression techniques. Although a penalized regression approach such as the LASSO or a more parsimonious model would have been methodologically preferable, the small number of events precluded reliable variable selection and penalization within this dataset; this constraint further underscores the hypothesis-generating nature of the present findings.

Another limitation concerns the timing of the MSPSS assessment. Perceived social support was measured during the postpartum follow-up rather than during pregnancy. Therefore, the temporal direction of the association between social support and PPD cannot be determined. Lower perceived social support may contribute to depressive symptoms, but depressive symptoms may also influence how mothers perceive and report the support available to them. Future longitudinal studies should measure social support both antenatally and postnatally to clarify whether it functions as a predictor, consequence, or bidirectional correlate of PPD.

Another limitation is the absence of a comparison group receiving conventional face-to-face postpartum follow-up. Therefore, this study cannot determine whether telepsychiatry is more effective, more acceptable, or superior to standard care. The findings should be interpreted as supporting the feasibility of a telepsychiatry-based postpartum mental health assessment rather than as demonstrating its comparative effectiveness. Future controlled studies are needed to compare the telepsychiatry-based follow-up with face-to-face or usual-care models.

From a methodological perspective, although all assessments were conducted by a single psychiatrist to maximize intra-rater consistency, the possibility of observer bias cannot be completely excluded. In addition, limiting the follow-up period to the first two months postpartum may have led to the omission of PPD cases that develop later.

Finally, since some of the psychosocial data were based on self-report scales, participants may have responded in a way influenced by social desirability bias. This study can be considered a pilot study due to its methodological design and the specific population examined. Future studies with longer follow-up periods, multicenter designs, and larger samples would strengthen the causal interpretability and the external validity of the findings.

## Figures and Tables

**Table 1:** Sociodemographic and psychosocial characteristics of the participants.

		*n*	%
Education level	≤12 years	37	63.8%
	>12 years	21	36.2%
Employment status	Unpaid work/housewife	40	69%
	Employed	18	31%
Social security coverage	Yes	56	96.6%
	No	2	3.4%
Economic status	Low	40	69%
	Good	18	31%
Infant’s sex	Girl	24	41.4%
	Boy	34	58.6%
Spouse’s education level	≤12 years	35	60.3%
	>12 years	23	39.7%
Spouse’s employment status	Unemployed	3	5.2%
	Employed	55	94.8%
Marital satisfaction	Low	20	34.5%
	High	38	65.5%
	Mean ± SD	Min–Max	Median
MSPSS-Total	66.90 ± 20.24	12–84	74
Significant Other	23.83 ± 6.94	4–28	28
Family	21.55 ± 7.92	4–28	26.5
Friends	21.52 ± 8.23	4–28	28
Age	30.12 ± 5.84	19–47	30
Gravidity	2.36 ± 1.48	1–6	2
Parity	1.91 ± 0.94	1–4	2

MSPSS: Multidimensional Scale of Perceived Social Support.

**Table 2:** Clinical and obstetric characteristics of the participants.

Variable		*n*	%
Comorbid medical condition	Yes	15	25.9
	No	43	74.1
Obesity	Yes	22	37.9
	No	36	62.1
Smoking	Yes	11	19
	No	47	81
Alcohol use	Yes	0	0
	No	58	100
History of psychiatric illness	Yes	19	32.8
	No	39	67.2
IVF pregnancy	Yes	5	8.6
	No	53	91.4
Preterm birth	Yes	5	8.6
	No	53	91.4
Advanced maternal age	Yes	11	19
	No	47	81
Fetal anomaly	Yes	11	19
	No	47	81
Complication during delivery	Yes	10	17.2
	No	48	82.8
Stressful life event during pregnancy	Yes	23	39.7
	No	35	60.3
Health problem during pregnancy	Yes	42	72.4
	No	16	27.6

IVF: In vitro fertilization.

**Table 3:** Cumulative detection of postpartum depression during the first two postpartum months.

Time Point	PPD+ (*n*)	%
Month 1	13	22.4%
Month 2 additional new-onset cases	6	10.3%
Cumulative total during first two months	19	32.7%

PPD+: Participants with postpartum depression.

**Table 4:** Longitudinal comparison of EPDS and MSPSS scores across PPD groups at months 1 and 2.

Variable	PPD− Median (Q1–Q3) [Min–Max]	Month-1 PPD+ Median (Q1–Q3) [Min–Max]	Month-2 PPD+ Median (Q1–Q3) [Min–Max]	H	*p*	PPD− vs. M1+ Z (*p*)	PPD− vs. M2+ Z (*p*)	M1+ vs. M2+ Z (*p*)
Month-1 EPDS Total	0 (0–3) [0–11]	17 (15–18) [13–23]	3 (0–6.75) [0–9]	33.068	<0.001	−5.608 (<0.001) †	−1.138 (0.255)	−3.437 (<0.001) †
MSPSS Total	84 (66–84) [17–84]	48 (29–58) [12–77]	65.5 (53.5–74.25) [49–78]	24.807	<0.001	−4.685 (<0.001) †	−2.476 (0.013) †	−2.326 (0.020)
MSPSS—Significant Other	28 (28–28) [8–28]	16 (7.5–23.5) [4–27]	22 (19.75–26.5) [19–28]	35.676	<0.001	−5.697 (<0.001) †	−3.608 (<0.001) †	−2.020 (0.043)
MSPSS—Family	28 (23–28) [4–28]	15 (10–18.5) [4–27]	19.5 (13.75–24.5) [13–26]	18.993	<0.001	−4.039 (<0.001) †	−2.684 (0.006) †	−0.970 (0.332)
MSPSS—Friends	28 (18–28) [4–28]	16 (5–22) [4–28]	21 (18.25–28) [16–28]	13.696	0.001	−3.650 (<0.001) †	−1.073 (0.272)	−1.806 (0.071)
Month-2 EPDS Total	0 (0–2) [0–11]	9 (4.5–13) [0–21]	15 (14–15.5) [14–17]	34.569	<0.001	−4.732 (<0.001) †	−4.485 (<0.001) †	−2.907 (0.004) †

EPDS: Edinburgh Postnatal Depression Scale; MSPSS: Multidimensional Scale of Perceived Social Support. Month-1 PPD+: participants diagnosed at the first-month assessment. Month-2 PPD+: participants with new-onset PPD detected at the second-month assessment who had no PPD diagnosis at month 1. Kruskal–Wallis test followed by pairwise Mann–Whitney U post hoc comparisons with Bonferroni correction (adjusted threshold: *p* < 0.017). † Statistically significant after Bonferroni correction.

**Table 5:** Comparison of sociodemographic and psychosocial characteristics between women with and without PPD.

		PPD+		PPD–		*p*	Z	Effect Size
		(*n*: 19)		(*n*: 39)				
Age	Mean ± SD	31.05 ± 6.74		29.67 ± 5.39		0.402 ^t^		Cohen’s d = 0.24
Gravidity	Median Min–Max	2 1–6		2 1–6		0.692	−0.396	r = 0.05
Parity	Median Min–Max	2 1–4		2 1–4		0.178	−1.346	r = 0.18
		%	*n*	%	*n*	*p*		
Education level	≤12 years	68.4%	13	64.9%	24	0.609		Cramer’s V = 0.07
	>12 years	31.6%	6	38.5%	15			
Employment status	Unpaid work/housewife	78.9%	15	64.1%	25	0.251		Cramer’s V = 0.15
	Employed	21.1%	4	35.9%	14			
Social security coverage	Yes	89.5%	17	100%	39	0.103 ^a^		Cramer’s V = 0.27
	No	10.5%	2	0%	0			
Economic status	Low	73.7%	14	66.7%	26	0.588		Cramer’s V = 0.07
	Good	26.3%	5	33.3%	13	0.938		
Infant’s sex	Girl	42.1%	8	41.0%	16			Cramer’s V = 0.01
	Boy	57.9%	11	59.0%	23			
Spouse’s education level	≤12 years	78.9%	15	51.3%	20	0.043 *		Cramer’s V = 0.27
	>12 years	21.1%	4	48.7%	19			
Spouse’s employment status	Unemployed	5.3%	1	5.1%	2	1 ^a^		Cramer’s V = 0.00
	Employed	94.7%	18	94.9%	37			
Marital satisfaction	Low	57.9%	11	23.1%	9	0.009 *		Cramer’s V = 0.34
	High	42.1%	8	76.9%	30			
		Median Min−Max		Median Min−Max		*p*	Z	
MSPSS-Total		51 12–78		84 17–84		<0.001 **	−4.776	r = 0.63
	Significant Other	19 4–28		28 8–28		<0.001 **	−5.823	r = 0.76
	Family	15 4–27		28 4–28		<0.001 **	−4.320	r = 0.57
	Friends	18 4–28		28 4–28		0.001 *	−3.322	r = 0.44

MSPSS: Multidimensional Scale of Perceived Social Support. PPD+: Participants with postpartum depression. PPD−: Participants without postpartum depression. ^t^ Independent sample *t* test; ^a^ Fisher’s Exact Test; Z: Mann–Whitney U test; Effect sizes are reported as Cohen’s d for normally distributed continuous variables, r for Mann–Whitney U tests, and Cramer’s V for categorical variables. * *p* < 0.05, ** *p* < 0.01.

**Table 6:** Comparison of clinical and obstetric characteristics between women with and without postpartum depression.

		PPD+ (*n*: 19)		PPD– (*n*: 39)			
		%	*n*	%	*n*	*p*	Effect Size Cramer’s V
Comorbid medical condition	Yes	31.6%	6	23.1%	9	0.533 ^a^	0.09
	No	68.4%	13	76.9%	30		
Obesity	Yes	31.6%	6	41%	16	0.486	0.09
	No	68.4%	13	59%	23		
Smoking	Yes	21.1%	4	17.9%	7	1 ^a^	0.04
	No	78.9%	15	82.1%	32		
History of psychiatric illness	Yes	36.8%	7	30.8%	12	0.644	0.06
	No	63.2%	12	69.2%	27		
IVF pregnancy	Yes	10.5%	2	7.7%	3	1 ^a^	0.05
	No	89.5%	17	92.3%	36		
Preterm birth	Yes	0%	0	12.8%	5	0.161 ^a^	0.21
	No	100%	19	87.2%	34		
Advanced maternal age	Yes	31.6%	6	12.8%	5	0.151 ^a^	0.22
	No	68.4%	13	87.2%	34		
Fetal anomaly	Yes	26.3%	5	15.4%	6	0.476 ^a^	0.13
	No	73.7%	14	84.6%	33		
Complication during delivery	Yes	21.1%	4	15.4%	6	0.714 ^a^	0.07
	No	78.9%	15	84.6%	33		
Stressful life event during pregnancy	Yes	42.1%	8	38.5%	15	0.790	0.03
	No	57.9%	11	61.5%	24		
Health problem during pregnancy	Yes	68.4%	13	74.4%	29	0.635	0.06
	No	31.6%	6	25.6%	10		

IVF: In vitro fertilization. PPD+: Participants with postpartum depression. PPD−: Participants without postpartum depression. ^a^ Fisher’s Exact Test; Z: Mann–Whitney U test; Effect sizes are reported as Cramer’s V for categorical variables.

**Table 7:** Logistic regression analysis of factors associated with postpartum depression.

Independent Variables	B	S.E.	Wald	*p*	OR (Exp(B))	95% CI
Marital Satisfaction	3.263	1.173	7.742	0.005 *	26.129	2.623–260.232
MSPSS total score	−0.106	0.032	11.226	<0.001 **	0.900	0.846–0.957
Fetal anomaly	2.714	1.340	4.103	0.043 *	15.095	1.092–208.666
Spouse’s education level	2.844	1.163	5.985	0.014 *	17.185	1.760–167.755
Comorbid medical condition	0.910	1.078	0.713	0.399	2.485	0.300–20.558
Stressful life event during pregnancy	−0.874	0.905	0.932	0.334	0.417	0.071–2.461
Constant	2.415	1.605	2.263	0.132	11.187	—

MSPSS: Multidimensional Scale of Perceived Social Support; * *p* < 0.05, ** *p* < 0.01.

## Data Availability

The datasets generated during and/or analyzed during the current study are available from the corresponding author on reasonable request.
